# How does working time impact perceived mental disorders? New insights into the U-shaped relationship

**DOI:** 10.3389/fpubh.2024.1402428

**Published:** 2024-07-03

**Authors:** Xiaoru Niu, Chao Li, Yuxin Xia

**Affiliations:** ^1^School of Mechanical, Electrical & Information Engineering, Shandong University, Weihai, China; ^2^Shenzhen Research Institute of Shandong University, Shenzhen, China; ^3^Business School, Shandong University, Weihai, China; ^4^Centre for Quality of Life and Public Policy Research, Shandong University, Qingdao, China; ^5^HSBC Business School, Peking University, Shenzhen, China

**Keywords:** working time, perceived mental disorders, depression, U-shaped relationship, instrumental variable approach

## Abstract

Based on a large-scale nationally representative survey in China, this paper uses the exogenous impact of automation on working hours as the instrumental variable to examine working time’s impact on perceived mental disorders, on the basis of dealing with endogeneity. Different from existing literature, it is found that the impact of working time on perceived mental disorders is U-shaped, rather than linear. Mental disorders firstly decrease with working hours. After working more than 48.688 h per week, further increases in working time carry notable mental health costs, leading to a positive relationship between working hours and depression. The turning point of this U-shaped relationship is almost in line with the International Labor Organization’s 48 working hours/week standard, justifying it from a mental health perspective. In addition, we further exclude the possibility of more complex nonlinear relationships between working time and perceived mental disorders. Furthermore, heterogeneities are found in the effects of working hours on mental disorders across different subgroups. Males are more depressed when working overtime. Older workers have a lower tolerance for overwork stress. The turning point is smaller for the highly educated group and they are more sensitive to working longer. Those with higher socioeconomic status are less depressed after exceeding the optimal hours of work. The increase in depression among rural workers faced with overwork is not prominent. Perceived mental disorders are lower among immigrants and those with higher health status. In addition, labor protection and social security help to weaken mental disorders caused by overtime work. In conclusion, this paper demonstrates that working time has a U-shaped impact on perceived mental disorders and highlights the vulnerability of certain groups, providing a reference for setting optimal working hours from a mental health perspective.

## 1 Introduction

Most countries have statutory limits of weekly working hours of 48 h or less which is the standard established in International Labor Organization (ILO) conventions (1). The rationality of this criterion lies in the fact that most of the literature on the relationship between working hours and mental health indicates that the two factors are negatively related, that is, the increase in working hours leads to perceived mental disorders (2–4). Policies to reduce excessive working hours are found to help improve people’s subjective welfare (5). For example, implementing policies that limit working hours can not only reduce chronic fatigue and burnout caused by overwork, but also enhance life satisfaction and happiness (6). Data can more intuitively reflect this relationship: compared with those who work 35–40 h per week, people working more than 55 h per week have a 1.74 times higher risk of depressive symptoms (7).

Existing research on work-life balance finds that excessively long working hours lead to health problems and reduce mental well-beings (8–11). Specifically, excessively long working hours reduce rest time, resulting in a heavier workload and increased work stress (12). This not only leads to various physical illnesses, such as stroke and coronary heart disease (13), but also increases the risk of anxiety and depression (14, 15). In addition, studies from the work arrangement perspective show that shift arrangements and night work increase depression, anxiety, cognitive impairment and even suicidal tendencies (16–19). This is because irregular working hours may disrupt social networks, leading to feelings of loneliness and isolation, which in turn increase mental stress (20). Moreover, the circadian rhythm disruption caused by night shifts could result in hormonal imbalances, decreased immunity, and other negative health consequences, exacerbating mental health issues (21, 22). Moreover, literature suggests that working hours moderate the relationship between life satisfaction and mental health, rather than exerting direct impacts (23). In addition, it is found that working time flexibility also plays a role and low flexibility is associated with worse mental health (24). More flexible working hours enable people to better balance work and life, thereby improving their overall mental health (25). For example, compared to traditional office settings, remote work offers greater flexibility and has been proven to significantly enhance job satisfaction (26, 27).

However, some studies indicate that the relationship between working time and mental disorders is not clear. A meta-analysis based on a sample of 21 studies finds a small but significant positive relationship between working time and mental health (28). There are also studies suggesting no significant correlation between working hours and mental health (29). Besides, mutual influence or reverse causality between the two factors is also assumed (30). For example, evidence indicates that mental health affects working hours through working motivation and absence (31). Specifically, individuals with poor mental health have reduced work motivation and increased absenteeism, leading to shorter working hours (32). Moreover, cultural backgrounds may influence the relationship between these two. For example, in some cultures that highly value work, long working hours may be considered normal and may not significantly affect mental health (33, 34).

Furthermore, heterogeneities in the relationship between working hours and mental health are documented in the literature. For example, studies have found that working overtime is more prominently associated with poor mental health in men, while less in women (35). This gender difference may be due to the social pressure on men to be the primary breadwinners, which causes them to bear greater financial responsibility and psychological stress when working long hours (36). Additionally, disparities are also reflected in groups with different socioeconomic statuses, educational levels and occupational skills. Those who are higher educated and more satisfied with living conditions are less negatively impacted on their mental health by overtime working (37, 38). For example, individuals with higher socioeconomic status are more likely to have access to superior resources and more effective social support to cope with the stress of long working hours (39). Nevertheless, another study shows that people with higher occupational skills experience greater perceived mental disorders due to long working hours (40). This means that when analyzing the relationship between working time and mental disorders, more attention needs to be paid to the heterogeneities among different subgroups.

From the above review, we find that existing research results support either a linear negative or positive correlation between working time and mental disorders. At the same time, some scholars believe that there is a problem of reverse causality in the relationship and thus it is difficult to determine the causality between working hours and mental health. The aim of this paper is to clarify the exact relationship between working time and mental disorders. Specifically, based on data from a large-scale, nationally representative survey in China, this paper investigates the effect of working hours on depression, using the exogenous impact of automation on working time as the instrumental variable. Additionally, we examine potential nonlinear relationships by including higher-order terms of working hours in the regressions and perform a series of robustness tests. Moreover, heterogeneities are investigated across various aspects, including gender, age, education, socioeconomic status, region, migration status, health condition, labor protection, and social security.

## 2 Materials and methods

### 2.1 Data source

Data used in this paper are from a large-scale nationally representative survey, Chinese General Social Survey (CGSS), conducted in 2017 and 2018 CGSS is one of the most important national, comprehensive and continuous academic survey projects in China, and is a member of the world General Social Survey (GSS) family. It collects extensive data at multiple levels and across various domains. CGSS aims to gather information on Chinese society to monitor and explain trends in work, behaviors, health and attitudes to examine the structure and functioning of society in general. CGSS sample covers 28 provinces/municipalities/autonomous regions in China and uses the multi-stage stratified Probability Proportionate to Size (PPS) sampling method, making it highly representative. Details of the study protocol are introduced in the Supplementary material and data files are available on http://cgss.ruc.edu.cn/English/Home.htm.

### 2.2 Measures

**Figure 1:**
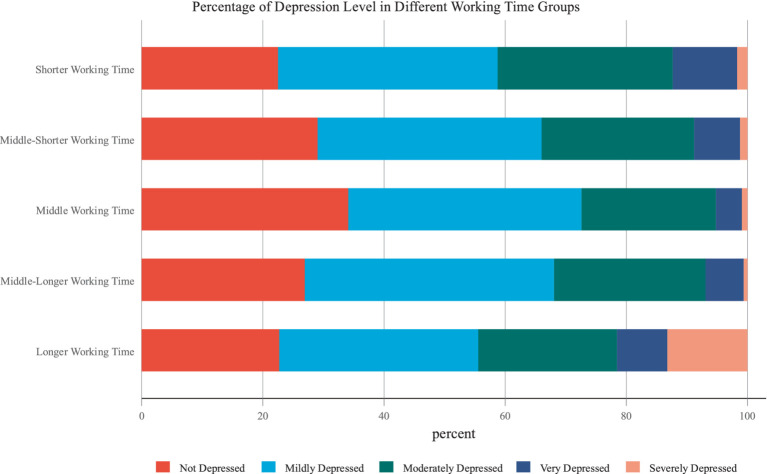
Relationship between working time and depression.

The explained variable in this paper is the depression degree, which is based on the classic Likert scale from 1 to 5 to characterize the level of depression. Specifically, respondents answer whether their current mental health status is not depressed, mildly depressed, moderately depressed, very depressed or severely depressed. This indicator is one of the most commonly used indexes to measure depression in the existing literature (41–44). The explanatory variable is the number of hours worked per week. We preliminarily analyze the relationship between depression and working time, by averagely dividing the sample according to the quantiles of working hours into five subsamples, including groups of shorter working time, middle-shorter working time, middle working time, middle-longer working time and longer working time. Figure 1 illustrates that the percentage of people who are not depressed and a little depressed in the middle working time group is significantly higher than that in other groups, while in this group very depressed and extremely depressed respondents account for the lowest percentage. The share of the very depressed and extremely depressed increases notably both at the upper and lower sides of the middle working time group. This pattern suggests that the middle working hours group experiences the lowest levels of depression and the relationship between working time and perceived mental disorders tends to be nonlinear.

Referring to the literature concerning depression (45–56), in order to avoid the omitted variables bias, we fully control factors affecting depression in the following five aspects. (1) Basic demographic characteristics include age, the quadratic term of age, gender, education level, health status and whether the respondent is a migrant. (2) Social characteristics include whether the respondent’s Hukou is in urban,^1^ and whether she/he belongs to ethnic minorities, is a religious believer and Communist Party of China (CPC) member. (3) Working characteristics include the logarithm of income, the respondent’s overall socio-economic status and whether she/he has pension and medical insurance. (4) Family characteristics include whether the respondent is married, her/his number of children and number of houses. (5) Regional and year dummies. The descriptive statistics of above variables are shown in Supplementary Table S1.

### 2.3 Methods

It is almost impossible to conduct randomized controlled trials on working hours and to explore how the variations of working time affect depression. Therefore, it is very difficult to investigate the causal relationship between the two factors using observational data. The biggest challenge here is the reverse causality, where mental disorders also affect working hours (30, 31). To deal with this problem, we use the exogenous impact of automation on working time as the instrumental variable to examine the causal effect of working time on depression. In addition, to analyze the nonlinear relationship between the two factors, we include both working time and its quadratic term into regressions. Specifically, the following Two Stage Least Square (2SLS) statistical model is constructed.


$$\begin{array}{l} Working\_time_{i}=\alpha_{0}+\alpha_{1}Automation_{i}\\ +\alpha_{2}Automation\_squared_{i}+x_{i}^{\prime }\psi^{1}+\lambda_{t}+\theta_{p}+\varepsilon_{i} \end{array}$$

$$\begin{array}{l} Working\_time\_squared_{i}=\beta_{0}\\ +\beta_{1}Automation_{i}+\beta_{2}Automation\_squared_{i}+x_{i}^{\prime }\psi^{2}+\lambda_{t}+\theta_{p}+\epsilon_{i} \end{array}$$

$$\begin{array}{l} Depression_{i}=\gamma_{0}+\gamma_{1}\;Work\overset{⌢}{ing}\_time_{i}\\ +\gamma_{2}\;Working\_\overset{⌢}{time}\_squared_{i}+x_{i}^{\prime }\psi^{3}+\lambda_{t}+\theta_{p}+\mu_{i} \end{array}$$

In the model, Working_time and Working_time_squared represent working hour per week and its squared term, respectively. Automation is an indicator of the extent to which the work performed by the respondent is affected by automation. Automation_squared is the square of Automation . Depression represents the degree of depression. $x_{i}^{\prime }$ is a vector of control variables introduced above. $\lambda_{t}$ and $\theta_{p}$ are time and province fixed effects. We use the first two equations to perform first-stage estimations of working time and its square and obtain their fitted values. In the second stage, the third equation is regressed using estimates of working hours and its square to examine the nonlinear effect of working time on perceived mental disorders.

In this 2SLS model, the instrumental variables are Automation and its quadratic term. The automation index is constructed by Autor and Dorn (57), which is the most commonly used indicator to measure the degree of automation’s impact on working time (58). Studies have confirmed that the larger the extent to which work is replaced by automation, the less working time is required (59). Therefore, this instrumental variable satisfies the correlation prerequisite. At the same time, owing to the following reasons, it also meets the exogeneity condition. First, the replacement of jobs by automation is caused by exogenous technological progress, independent of individual-level characteristics of workers. Second, the Automation index is measured utilizing the task traits of different occupations in the Dictionary of Occupational Titles by the United States Department of Labor in 1977 (57). Because the individual status of a current worker could not affect the characteristics of the occupation in 1977, this instrumental variable well satisfies the exogeneity requirement, especially in terms of avoiding reverse causality. In Table 1, we show the relevant statistical test results for the instrumental variables. Therefore, based on the above 2SLS model, we can use the exogenous changes brought about by automation to scientifically examine how working hours affect perceived mental disorders on the basis of tackling endogeneity.

**Table 1:** Empirical results.

Model	(1) 2SLS	(2) 2SLS	(3) 2SLS	(4) 2SLS	(5) 2SLS	(6) 2SLS	(7) 2SLS
Variable	Depression	Depression	Depression	Depression	Depression	Depression	Depression
Working_time	−0.0648^***^ (0.0240)	−0.0631^***^ (0.0185)	−0.0652^***^ (0.0192)	−0.0843^***^ (0.0222)	−0.0852^***^ (0.0221)	−0.0736^***^ (0.0235)	−0.0498 (0.1581)
Working_time_squared	0.0007^**^ (0.0003)	0.0007^***^ (0.0002)	0.0007^***^ (0.0002)	0.0009^***^ (0.0002)	0.0009^***^ (0.0002)	0.0008^***^ (0.0002)	0.0001 (0.0043)
Working_time_cubed							0.0000 (0.0000)
Age		0.0002 (0.0071)	0.0014 (0.0069)	0.0025 (0.0069)	0.0129^*^ (0.0071)	0.0133^*^ (0.0069)	0.0135^**^ (0.0067)
Age_squared		−0.0001 (0.0001)	−0.0001 (0.0001)	−0.0001 (0.0001)	−0.0002^**^ (0.0001)	−0.0002^**^ (0.0001)	−0.0002^**^ (0.0001)
Whether female		0.0557^*^ (0.0299)	0.0491 (0.0310)	0.0686^**^ (0.0304)	0.0757^**^ (0.0301)	0.0800^***^ (0.0303)	0.0728 (0.0462)
Education level		0.0102 (0.0101)	0.0144^*^ (0.0083)	0.0166^**^ (0.0071)	0.0175^**^ (0.0070)	0.0173^***^ (0.0066)	0.0147 (0.0166)
Health status		−0.3019^***^ (0.0147)	−0.2999^***^ (0.0147)	−0.2840^***^ (0.0150)	−0.2813^***^ (0.0149)	−0.2789^***^ (0.0151)	−0.2792^***^ (0.0154)
Whether migrants		0.0112 (0.0313)	0.0137 (0.0329)	0.0226 (0.0330)	0.0091 (0.0327)	0.0608^*^ (0.0330)	0.0659 (0.0411)
Whether Hukou in urban			0.0004 (0.0348)	0.0026 (0.0318)	0.0009 (0.0311)	0.0307 (0.0300)	0.0258 (0.0423)
Whether ethnic minorities			−0.0554 (0.0400)	−0.0846^*^ (0.0436)	−0.0876^**^ (0.0434)	−0.1802^***^ (0.0483)	−0.1746^***^ (0.0616)
Whether religious believer			0.0486 (0.0322)	0.0487 (0.0351)	0.0441 (0.0355)	0.0463 (0.0355)	0.0423 (0.0427)
Whether CPC member			−0.0843^***^ (0.0312)	−0.0770^**^ (0.0332)	−0.0649^*^ (0.0336)	−0.0697^**^ (0.0327)	−0.0771 (0.0564)
ln_Income				0.0359^**^ (0.0150)	0.0362^**^ (0.0147)	0.0339^**^ (0.0152)	0.0311 (0.0259)
Socio-economic status				−0.1568^***^ (0.0213)	−0.1504^***^ (0.0215)	−0.1490^***^ (0.0207)	−0.1508^***^ (0.0224)
Whether having pension				−0.0137 (0.0261)	−0.0096 (0.0264)	−0.0180 (0.0257)	−0.0241 (0.0443)
Whether having medical insurance				0.0465 (0.0450)	0.0646 (0.0454)	0.0529 (0.0435)	0.0540 (0.0437)
Whether married					−0.1445^***^ (0.0302)	−0.1340^***^ (0.0289)	−0.1309^***^ (0.0336)
Family size					−0.0020 (0.0073)	−0.0070 (0.0073)	−0.0062 (0.0092)
Number of children					0.0126 (0.0121)	0.0053 (0.0112)	0.0061 (0.0119)
Number of houses					−0.0131 (0.0153)	−0.0055 (0.0155)	−0.0050 (0.0161)
Year dummy	No	No	No	No	No	Yes	Yes
Province dummies	No	No	No	No	No	Yes	Yes
Constant	3.3446^***^ (0.3634)	4.4161^***^ (0.2256)	4.4133^***^ (0.2555)	4.4720^***^ (0.2637)	4.3104^***^ (0.2743)	3.8939^***^ (0.3215)	3.7731^***^ (0.8921)
Observations	12,452	12,408	12,377	11,738	11,655	11,655	11,655

***, **, and * indicate significance at the levels of 1, 5, and 10%, respectively. The values in parentheses are standard errors robust to heteroskedasticity. Yes means the corresponding variables are controlled in the regression, while *N* means not controlled. The same for other tables in this paper and the Supplementary material.

The range of this indicator is from −6.190 to 4.235. According to this index, the Automation indicators of sales and service occupations, mining, construction, manufacturing, transportation workers, personal and protective service workers are equal to or close to 4.235. This means that people performing these occupations are more likely to be replaced by automation, thus their income is more negatively impacted by exogenous artificial intelligence technological progress. Conversely, the values for corporate managers, physical, mathematical, and engineering professionals, life sciences and health professionals, and others are equal to or close to the minimum of −6.190. This suggests that these occupations are complementary to automation applications, resulting in a positive impact on their income from automation technology. A detailed description of the index is provided in the Supplementary material.

## 3 Results

### 3.1 Benchmark results

Estimation results are shown in Table 1. Column (1) is the regression result without controlling any variables. It is shown that the estimates of both working time and its squared term are statistically significant. The estimated coefficient of working hours is significantly negative, while that of its square is positive. This means that working time’s effect on depression is nonlinear. In the regressions from columns (2) to (6), we gradually add controls of the demographic characteristics, social characteristics, working characteristics, family background and regional and time dummies. With the inclusion of different types of controls, estimated coefficients of Working_time and Working_time_squared are all statistically significant at the 1% level. This means that the nonlinear relationship between working time and depression is very robust.

**Figure 2:**
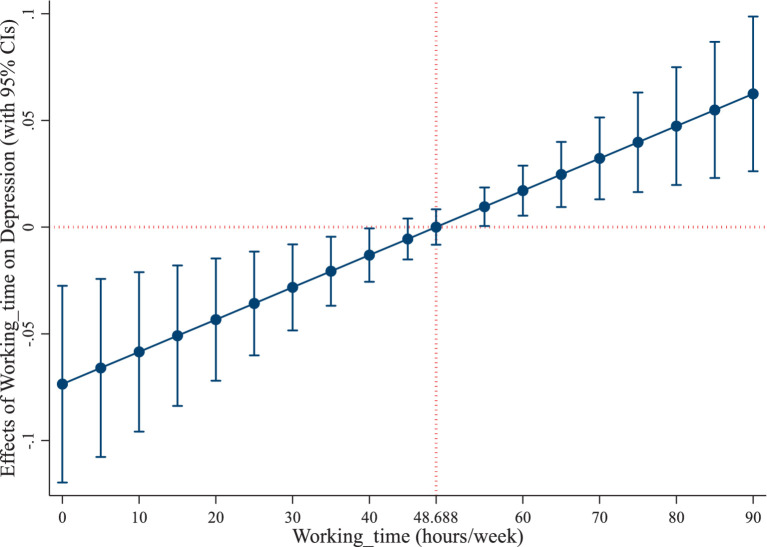
U-shaped effect of working time on depression.

As shown in column (6), the estimates of working time and its squared term are −0.0736 and 0.0008. This means that when working hours are lower than 48.688 h per week (=0.0735557/(0.0007554*2)), depression reduces as working time increases. This is mainly due to the fact that when working time is low, income rises with longer working hours, which is conducive to reducing perceived mental disorders. Existing literature provides indirect explanations concerning why working longer during the lower working time interval could help reduce perceived mental disorders. A study from the United Kingdom found that for unemployed or economically inactive individuals, increasing their working time can improve their social participation and mental health (60). However, after working hours exceed the turning point of 48.688 h per week, depression increases with working longer. This turning point is almost in line with the International Labor Organization (ILO) standard of working no more than 48 h per week (61). Therefore, this research justifies the standard from a mental health perspective. Figure 2 intuitively illustrates that as working time increases, its effect on depression is firstly negative and then positive, meaning a U-shaped effect of working time on depression. In CGSS, 23.090% of the respondents work more than 48.688 h per week. This means that from the perspective of mental health, almost 1 in 4 people suffer from rising depression caused by overwork.

In addition, we further examine whether the relationship between working hours and depression is a U-shaped relationship or a more complex association with more turning points. We include working time and its quadratic and cubic terms into the instrumental variable model. Regression results are shown in column (7) of Table 1. It is demonstrated that all of the three variables are not significant. This means that the multicollinearity among them causes overfitting. Therefore, the cubic term of working time should not be included in the regression. This confirms that the relationship between working hours and perceived mental disorders is not cubic or nonlinear with more turning points.

### 3.2 Heterogeneities

**Figure 3:**
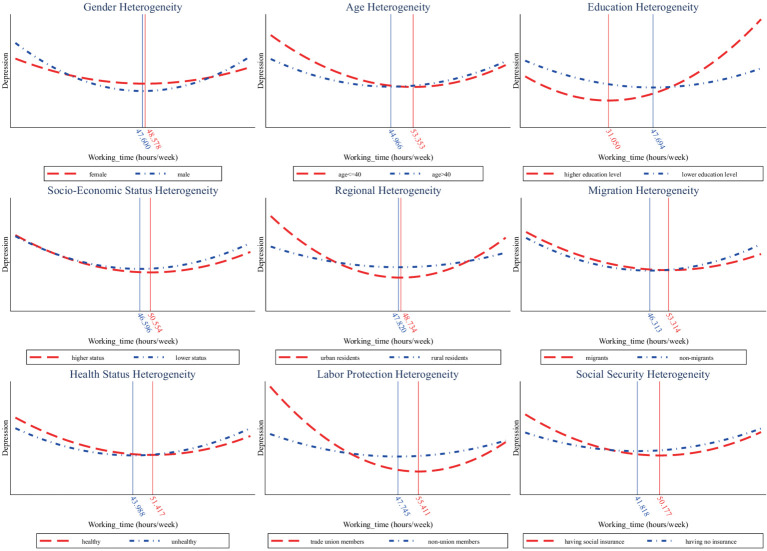
Heterogeneities in the U-shaped impact of working time on depression.

This paper further investigates the heterogeneities of working time’s U-shaped effects on depression from multiple perspectives, illustrated in Figure 3. Here, we mainly focus on the turning points and the disparities in perceived mental disorders among different groups. In terms of gender, it is shown that the turning points for men and women are very close. However, men suffer more perceived mental disorders from overwork than women. With regard to age, older workers’ turning point is much smaller than that of young workers (44.966 < 53.353). Additionally, older workers experience higher levels of depression when faced with overwork. In respect of educational heterogeneity, it is found that the turning point is smaller for the higher educated group with a college degree or above, meaning that they are more sensitive to working hours and less willing to work longer. Nevertheless, when working time does not exceed the turning point, their depression level is lower, which may be attributed to their better working environment.

As to socioeconomic status, the higher status group experiences lower levels of depression when working longer than the optimal hours. Regarding regional differences, the U-shaped curve of rural residents is flatter and especially after the turning point, the upward trend is not prominent. This is related to the relatively lower living standards of residents in rural areas under China’s urban–rural dual economic structure, in which rural workers are more willing to work more hours for higher income. In addition, findings suggest that migrants and healthier people have a greater turning point in the U-shaped relationship between working hours and depression. When the optimal working time is exceeded, perceived mental disorders are also lower among them, meaning that migrants and those with healthier status are more tolerant to overwork.

Furthermore, we also pay special attention to whether labor protection and social security can alleviate the adverse effects of overwork on depression. In terms of labor protection, we compare the difference between trade union members and nonunion members. It is found that union members have a larger turning point and they consistently experience lower depression than non-union members as working time increases. This suggests that labor protection from unions can help to mitigate the negative impact of overtime on mental health. Besides, we explore the heterogeneity in terms of social security and find similar results. Those with social insurance have a higher turning point and experience lower perceived mental disorders when working overtime, meaning that social security also helps to reduce its side effect on mental health.

### 3.3 Robustness checks

We conduct a series of robustness checks on the U-shaped relationship between working time and perceived mental disorders. First, the sample sizes are different from columns (1) to (6). This is because the number of observations for different control variables differs slightly. As more control variables are included in the regressions, the sample size decreases to some extent. Naturally, we are concerned about whether this affects the results of this paper. Therefore, we conduct a robustness test, with the results presented in Supplementary Table S2. Second, another indicator measuring the impact of automation is used as the instrumental variable in the 2SLS model. Details of this IV are explained in Supplementary Table S3, in which the statistical results are almost identical with the benchmark regressions, lending further credence to the robustness of the conclusion. Third, “whether feeling depressed” is regressed as the explained variable using the IV-Probit model. Results of Supplementary Table S4 show that both working time and its quadratic term have significant effects on this dependent variable, further confirming the U-shaped relationship between working hours and perceived mental disorders.

Fourth, we perform regressions using other types of instrumental variables models, including limited information maximum likelihood estimation (LIML), generalized method of moments (GMM) and efficient two-step GMM and iterative GMM. Supplementary Table S5 demonstrates that regardless of which instrumental variable estimation method is used, results are almost the same with benchmark estimations. Fifth, considering that the nonlinear relationship between working time and depression has not been paid attention to in the literature, we specifically examine whether the square of working time is an important factor for predicting depression. In this regard, we use machine learning methods such as Lasso, Ridge, and Elastic Net models to perform penalized regression. Supplementary Table S6 demonstrates that working_time_squared is one of the key predictors of perceived mental disorders in all these models. This further confirms the robustness of nonlinear relationship between working time and depression. Sixth, a placebo test of working hours is performed to check the endogeneity after using the IV method. As illustrated and explained in Supplementary Figure S1, the U-shaped relationship between working hours and depression is not caused by other omitted factors.

## 4 Conclusion

Using a large-scale nationally representative survey in China, this paper examines the nonlinear impact of working hours on perceived mental disorders. Applying the exogenous impact of automation on working time as the instrumental variable, we investigate the causal relationship between working time and perceived mental disorders on the basis of dealing with endogeneity. It is found that the effect of working time on depression is U-shaped, rather than linear. When working less than 48.688 h per week, depression reduces as working hours increase. This may be because within this range, increased working hours could enhance an individual’s sense of self-worth, maintain moderate social interactions, and usually lead to higher income (6). These factors contribute to improved mental health. However, after exceeding this threshold, the mental health costs outweigh the benefits aforementioned, resulting in a positive correlation between working hours and depression. Long working hours result in chronic fatigue and sleep deprivation, increase psychological stress, and weaken social support systems, thereby having a significant negative impact on physical and mental health (12, 14). These findings justify the ILO’s working time standard of not working more than 48 h per week. This underscores the importance of reasonably arranging work hours to ensure workers’ health and safety, avoiding both excessively long and short working hours. The nonlinear causal relationship is a contribution to the existing literature in which the linear correlation between working time and depression is assumed (62–66). Based on this, we further find that there are no more complex nonlinear relationships with more turning points between working hours and mental health. Moreover, various robustness tests are carried out, all of which support the above conclusions.

Heterogeneity analysis shows that when working hours exceed the turning point, men’s depression level is higher. Older workers have a lower tolerance for overtime working stress. The turning point of the higher educated group is smaller and they are less willing to work longer. Those with higher socioeconomic status experience lower levels of depression after exceeding optimal working hours. The increasing levels of depression among rural residents are not pronounced, meaning that they are more willing to work more to raise income. After exceeding the turning point, perceived mental disorders are lower among migrants and those with better health conditions. In addition, it is also discovered that labor protection and social security can help to reduce mental disorders caused by overwork. Analytical results in this paper imply that the optimal working hours should be considered from a mental health perspective and different turning points of the U-shaped relationship among different groups should be noted. In addition, heterogeneities imply that some disadvantaged subgroups suffer from more perceived mental disorders due to excessive working and therefore need to be given more attention.

## Data availability statement

The original contributions presented in the study are included in the article/Supplementary material, further inquiries can be directed to the corresponding author.

## Ethics statement

The studies involving human participants were reviewed and approved by the Institutional Review Board, Renmin University of China. The participants provided their written informed consent to participate in the survey.

## Author contributions

XN: Conceptualization, Formal analysis, Methodology, Software, Writing – original draft, Writing – review & editing. CL: Conceptualization, Data curation, Methodology, Project administration, Supervision, Writing – original draft, Writing – review & editing. YX: Formal analysis, Investigation, Software, Visualization, Writing – review & editing.

## Footnotes

^1^Hukou is a system of household registration used in mainland China, mainly identifying a person as a rural or urban resident.
