# Supplementary material for: How does working time impact perceived mental disorders? New insights into the U-shaped relationship
Source: Front Public Health. 2024 Jul 3;12:1402428. doi: 10.3389/fpubh.2024.1402428 (PMC11252037; doi:10.3389/fpubh.2024.1402428)
Supplement: Supplementary file 1 [file Data_Sheet_1.pdf]

Supplementary Material

Supplementary Table 1 Descriptive statistics

Variable	Obs	Mean	Std. Dev.	Min	Max
Dependent Variable					
Depression	12452	2.153	0.956	1	5
Explanatory Variables					
Working_time	12476	43.900	23.468	0.000	88.000
Working_time_squared	12476	2477.896	2039.450	0.000	7744.000
Instrument Variables					
Automation	12476	-0.471	1.334	-6.190	4.235
Automation_squared	12476	2.000	4.179	0.000	38.314
Demographic Characteristics					
Age	12476	45.409	13.143	18.000	75.000
Age_squared	12476	2234.706	1219.873	324.000	5625.000
Whether female	12476	0.471	0.499	0	1
Education level	12472	5.497	3.408	1	13
Health status	12472	3.686	1.027	1	5
Whether migrants	12440	0.157	0.363	0	1
Social Characteristics					
Whether Hukou in urban	12451	0.302	0.459	0	1
Whether ethnic minorities	12476	0.081	0.272	0	1
Whether religious believer	12476	0.096	0.294	0	1
Whether CPC member	12461	0.101	0.302	0	1
Working Characteristics					
ln_Income	11913	9.450	2.774	0.000	16.113
Socio-economic status	12380	0.497	0.500	0	1
Whether having pension	12459	0.742	0.438	0	1
Whether having medical insurance	12470	0.934	0.248	0	1
Family Characteristics					
Whether married	12476	0.805	0.396	0	1
Family size	12463	2.950	1.525	1	10
Number of children	12456	1.524	1.093	0	8
Number of houses	12374	1.118	0.658	0	11
Year dummy					
Province dummies					

Notes: The meanings of dummy variables are all “Yes=1, No=0”. The education level is classified from 1 to 13: 1-without any education, 2-kindergarten, 3-primary school, 4-junior high school, 5-vocational high school, 6-ordinary high school, 7-techinical secondary school, 8-technical high school, 9-junior college (adult higher education), 10-junior college (regular higher education), 11-undergraduate (adult higher education), 12-undergraduate (regular higher education), 13-postgraduate and above. Health status is based on the classic Likert scale from 1 to 5 to represent 1-unhealthy, 2-relatively unhealthy, 3-general, 4-relatively healthy, 5-healthy.

Supplementary Table 2. Robustness checks using consistent samples

Model<br>Variable	(1) 2SLS<br>Depression	(2) 2SLS<br>Depression	(3) 2SLS<br>Depression	(4) 2SLS<br>Depression	(5) 2SLS<br>Depression	(6) 2SLS<br>Depression	(7) 2SLS<br>Depression
Working_time	-0.0837***	-0.0745***	-0.0762***	-0.0858***	-0.0852***	-0.0736***	-0.0498
	(0.0278)	(0.0201)	(0.0207)	(0.0222)	(0.0221)	(0.0235)	(0.1581)
Working_time_ squared	0.0009***	0.0008***	0.0008***	0.0009***	0.0009***	0.0008***	0.0001
	(0.0003)	(0.0002)	(0.0002)	(0.0002)	(0.0002)	(0.0002)	(0.0043)
Working_time_ cubed							0.0000
							(0.0000)
Age		0.0040	0.0054	0.0033	0.0129*	0.0133*	0.0135**
		(0.0079)	(0.0077)	(0.0070)	(0.0071)	(0.0069)	(0.0067)
Age_squared		-0.0001	-0.0001	-0.0001	-0.0002**	-0.0002**	-0.0002**
		(0.0001)	(0.0001)	(0.0001)	(0.0001)	(0.0001)	(0.0001)
Whether female		0.0560*	0.0482	0.0715**	0.0757**	0.0800***	0.0728
		(0.0323)	(0.0333)	(0.0303)	(0.0301)	(0.0303)	(0.0462)
Education level		0.0155	0.0177**	0.0170**	0.0175**	0.0173***	0.0147
		(0.0110)	(0.0088)	(0.0071)	(0.0070)	(0.0066)	(0.0166)
Health status		-0.2913***	-0.2901***	-0.2829***	-0.2813***	-0.2789***	-0.2792***
		(0.0165)	(0.0165)	(0.0151)	(0.0149)	(0.0151)	(0.0154)
Whether migrants		0.0281	0.0278	0.0188	0.0091	0.0608*	0.0659
		(0.0331)	(0.0346)	(0.0331)	(0.0327)	(0.0330)	(0.0411)
Whether Hukou in urban			0.0124	0.0045	0.0009	0.0307	0.0258
			(0.0375)	(0.0322)	(0.0311)	(0.0300)	(0.0423)
Whether ethnic minorities			-0.0810*	-0.0860*	-0.0876**	-0.1802***	-0.1746***
			(0.0429)	(0.0439)	(0.0434)	(0.0483)	(0.0616)
Whether religious believer			0.0474	0.0456	0.0441	0.0463	0.0423
			(0.0345)	(0.0355)	(0.0355)	(0.0355)	(0.0427)
Whether CPC member			-0.0788**	-0.0752**	-0.0649*	-0.0697**	-0.0771
			(0.0332)	(0.0335)	(0.0336)	(0.0327)	(0.0564)
ln_Income				0.0362**	0.0362**	0.0339**	0.0311
				(0.0150)	(0.0147)	(0.0152)	(0.0259)
Socio-economic status				-0.1569***	-0.1504***	-0.1490***	-0.1508***
				(0.0214)	(0.0215)	(0.0207)	(0.0224)
Whether having pension				-0.0108	-0.0096	-0.0180	-0.0241
				(0.0264)	(0.0264)	(0.0257)	(0.0443)
Whether having medical insurance				0.0537	0.0646	0.0529	0.0540
				(0.0455)	(0.0454)	(0.0435)	(0.0437)
Whether married					-0.1445***	-0.1340***	-0.1309***
					(0.0302)	(0.0289)	(0.0336)
Family size					-0.0020	-0.0070	-0.0062
					(0.0073)	(0.0073)	(0.0092)
Number of children					0.0126	0.0053	0.0061
					(0.0121)	(0.0112)	(0.0119)
Number of houses					-0.0131	-0.0055	-0.0050
					(0.0153)	(0.0155)	(0.0161)
Year dummy	No	No	No	No	No	Yes	Yes
Province dummies	No	No	No	No	No	Yes	Yes
Constant	3.6412***	4.5021***	4.5053***	4.4626***	4.3104***	3.8939***	3.7731***
	(0.4184)	(0.2392)	(0.2686)	(0.2637)	(0.2743)	(0.3215)	(0.8921)
Observations	11655	11655	11655	11655	11655	11655	11655

Supplementary Table 3. 2SLS regression results using another automation index as the instrument variable

Model Variable	(1) 2SLS Depression	(2) 2SLS Depression	(3) 2SLS Depression	(4) 2SLS Depression	(5) 2SLS Depression	(6) 2SLS Depression	(7) 2SLS Depression
Working_time	-0.0618** (0.0248)	-0.0600*** (0.0188)	-0.0621*** (0.0194)	-0.0810*** (0.0221)	-0.0820*** (0.0220)	-0.0704*** (0.0234)	-0.0554 (0.1570)
Working_time_squared	0.0006** (0.0003)	0.0007*** (0.0002)	0.0007*** (0.0002)	0.0008*** (0.0002)	0.0009*** (0.0002)	0.0007*** (0.0002)	0.0003 (0.0042)
Working_time_cubed							0.0000 (0.0000)
Age		-0.0011 (0.0070)	0.0002 (0.0069)	0.0016 (0.0068)	0.0121* (0.0070)	0.0125* (0.0068)	0.0126* (0.0066)
Age_squared		-0.0001 (0.0001)	-0.0001 (0.0001)	-0.0001 (0.0001)	-0.0002** (0.0001)	-0.0002** (0.0001)	-0.0002** (0.0001)
Whether female		0.0616** (0.0296)	0.0552* (0.0307)	0.0732** (0.0299)	0.0799*** (0.0297)	0.0847*** (0.0299)	0.0804* (0.0444)
Education level		0.0093 (0.0104)	0.0139 (0.0084)	0.0165** (0.0071)	0.0174** (0.0070)	0.0174*** (0.0066)	0.0158 (0.0166)
Health status		-0.3041*** (0.0147)	-0.3021*** (0.0147)	-0.2858*** (0.0148)	-0.2830*** (0.0148)	-0.2807*** (0.0149)	-0.2809*** (0.0155)
Whether migrants		0.0067 (0.0311)	0.0088 (0.0326)	0.0189 (0.0327)	0.0059 (0.0323)	0.0578* (0.0327)	0.0610 (0.0403)
Whether Hukou in urban			-0.0032 (0.0350)	0.0008 (0.0317)	-0.0008 (0.0311)	0.0292 (0.0299)	0.0261 (0.0429)
Whether ethnic minorities			-0.0517 (0.0395)	-0.0813* (0.0429)	-0.0845** (0.0427)	-0.1773*** (0.0476)	-0.1736*** (0.0618)
Whether religious believer			0.0480 (0.0319)	0.0493 (0.0347)	0.0445 (0.0350)	0.0470 (0.0351)	0.0446 (0.0420)
Whether CPC member			-0.0849*** (0.0310)	-0.0770** (0.0328)	-0.0651* (0.0333)	-0.0698** (0.0325)	-0.0745 (0.0560)
ln_Income				0.0333** (0.0148)	0.0338** (0.0146)	0.0315** (0.0150)	0.0296 (0.0264)
Socio-economic status				-0.1558*** (0.0210)	-0.1497*** (0.0212)	-0.1483*** (0.0205)	-0.1495*** (0.0223)
Whether having pension				-0.0135 (0.0258)	-0.0097 (0.0261)	-0.0175 (0.0254)	-0.0213 (0.0439)
Whether having medical insurance				0.0467 (0.0444)	0.0648 (0.0449)	0.0530 (0.0430)	0.0537 (0.0432)
Whether married					-0.1456*** (0.0298)	-0.1351*** (0.0285)	-0.1331*** (0.0330)
Family size					-0.0018 (0.0072)	-0.0068 (0.0072)	-0.0062 (0.0092)
Number of children					0.0131 (0.0119)	0.0053 (0.0110)	0.0059 (0.0119)
Number of houses					-0.0123 (0.0152)	-0.0045 (0.0154)	-0.0041 (0.0161)
Year dummy	No	No	No	No	No	Yes	Yes
Province dummies	No	No	No	No	No	Yes	Yes
Constant	3.2871*** (0.3720)	4.3733*** (0.2253)	4.3668*** (0.2547)	4.4304*** (0.2609)	4.2691*** (0.2712)	3.8487*** (0.3187)	3.7710*** (0.8989)

Observations	12452	12408	12377	11738	11655	11655	11655

Note: The automation index used in this paper is calculated based on the Standard Occupational Classification (SOC) 2009 system. To be used in CGSS, it is needed to convert this index to the International Standard Classification of Occupations 2008 (ISCO-2008) indicator using the occupational crosswalk system from the United States Department of Labor.

Supplementary Table 4. IV-Probit regression results using Whe_depression as the dependent variable

Model<br>Variable	(1)<br>IV-Probit<br>Whe_<br>depression	(2)<br>IV-Probit<br>Whe_<br>depression	(3)<br>IV-Probit<br>Whe_<br>depression	(4)<br>IV-Probit<br>Whe_<br>depression	(5)<br>IV-Probit<br>Whe_<br>depression	(6)<br>IV-Probit<br>Whe_<br>depression	(7)<br>IV-Probit<br>Whe_<br>depression
num_workhour	-0.0778***	-0.0728***	-0.0745***	-0.0856***	-0.0849***	-0.0811***	0.0450
	(0.0211)	(0.0185)	(0.0187)	(0.0176)	(0.0178)	(0.0210)	(0.2005)
num_workhour_2	0.0008***	0.0008***	0.0008***	0.0009***	0.0009***	0.0009***	-0.0026
	(0.0003)	(0.0002)	(0.0002)	(0.0002)	(0.0002)	(0.0002)	(0.0053)
num_workhour_3							0.0000
							(0.0000)
Age		-0.0013	-0.0011	-0.0003	0.0112	0.0114	0.0127
		(0.0093)	(0.0090)	(0.0082)	(0.0078)	(0.0080)	(0.0079)
Age_squared		-0.0001	-0.0001	-0.0001	-0.0002**	-0.0002**	-0.0002**
		(0.0001)	(0.0001)	(0.0001)	(0.0001)	(0.0001)	(0.0001)
Whether female		0.0840*	0.0757*	0.1000**	0.1054**	0.1116**	0.0741
		(0.0428)	(0.0440)	(0.0422)	(0.0419)	(0.0463)	(0.0669)
Education level		0.0188	0.0195**	0.0198***	0.0197***	0.0173**	0.0036
		(0.0122)	(0.0097)	(0.0076)	(0.0075)	(0.0077)	(0.0216)
Health status		-0.3464***	-0.3424***	-0.3104***	-0.3101***	-0.3171***	-0.3221***
		(0.0459)	(0.0470)	(0.0488)	(0.0483)	(0.0533)	(0.0512)
Whether migrants		0.0833**	0.0908**	0.0853**	0.0783**	0.0897**	0.1181**
		(0.0382)	(0.0388)	(0.0364)	(0.0368)	(0.0414)	(0.0478)
Whether Hukou in urban			0.0301	0.0266	0.0251	0.0490	0.0237
			(0.0441)	(0.0374)	(0.0368)	(0.0362)	(0.0564)
Whether ethnic minorities			-0.0481	-0.0686	-0.0739	-0.1660***	-0.1380*
			(0.0478)	(0.0472)	(0.0470)	(0.0548)	(0.0832)
Whether religious believer			0.0868**	0.0869**	0.0758*	0.0681	0.0474
			(0.0394)	(0.0416)	(0.0414)	(0.0442)	(0.0559)
Whether CPC member			-0.0640	-0.0527	-0.0438	-0.0484	-0.0885
			(0.0443)	(0.0431)	(0.0435)	(0.0452)	(0.0696)
ln_Income				0.0332**	0.0325**	0.0325**	0.0181
				(0.0146)	(0.0145)	(0.0158)	(0.0339)
Socio-economic status				-0.1662***	-0.1661***	-0.1768***	-0.1887***
				(0.0306)	(0.0315)	(0.0348)	(0.0327)
Whether having pension				0.0078	0.0098	-0.0096	-0.0423
				(0.0306)	(0.0310)	(0.0324)	(0.0548)
Whether having medical insurance				0.0389	0.0549	0.0471	0.0539
				(0.0506)	(0.0512)	(0.0520)	(0.0525)
Whether married					-0.1705***	-0.1588***	-0.1437***
					(0.0418)	(0.0427)	(0.0531)
Family size					0.0053	-0.0022	0.0025
					(0.0089)	(0.0092)	(0.0119)
Number of children					0.0188	0.0101	0.0147
					(0.0151)	(0.0142)	(0.0148)
Number of houses					0.0111	0.0171	0.0201
					(0.0188)	(0.0203)	(0.0211)
Year dummy	No	No	No	No	No	Yes	Yes
Province dummies	No	No	No	No	No	Yes	Yes
Constant	0.9854***	2.1093***	2.1252***	2.0041***	1.7891***	1.6122***	0.9862
	(0.3431)	(0.1809)	(0.1947)	(0.1750)	(0.1975)	(0.2598)	(1.2016)
Observations	12452	12408	12377	11738	11655	11655	11655

Supplementary Table 5. Regression results using different instrument variable methods

Model	(1) First Stage	(2) First Stage	(3) 2SLS Second Stage	(4) LIML Second Stage	(5) GMM Second Stage	(6) IGMM Second Stage
Variable	Working_ time	Working_ time_squared	Depression	Depression	Depression	Depression
Working_time			-0.0736***	-0.0736***	-0.0736***	-0.0736***
			(0.0235)	(0.0235)	(0.0235)	(0.0235)
Working_time_ squared			0.0008***	0.0008***	0.0008***	0.0008***
			(0.0002)	(0.0002)	(0.0002)	(0.0002)
RII	-0.039	-38.881**				
	(0.164)	(15.598)				
RII_squared	0.548***	49.007***				
	(0.063)	(6.099)				
Age	0.677***	54.912***	0.0133*	0.0133*	0.0133*	0.0133*
	(0.117)	(10.003)	(0.0069)	(0.0069)	(0.0069)	(0.0069)
Age_squared	-0.011***	-0.857***	-0.0002**	-0.0002**	-0.0002**	-0.0002**
	(0.001)	(0.106)	(0.0001)	(0.0001)	(0.0001)	(0.0001)
Whether female	-5.053***	-468.058***	0.0800***	0.0800***	0.0800***	0.0800***
	(0.418)	(36.515)	(0.0303)	(0.0303)	(0.0303)	(0.0303)
Education level	-0.731***	-94.278***	0.0173***	0.0173***	0.0173***	0.0173***
	(0.083)	(7.437)	(0.0066)	(0.0066)	(0.0066)	(0.0066)
Health status	0.928***	51.233**	-0.2789***	-0.2789***	-0.2789***	-0.2789***
	(0.237)	(20.535)	(0.0151)	(0.0151)	(0.0151)	(0.0151)
Whether migrants	3.249***	312.493***	0.0608*	0.0608*	0.0608*	0.0608*
	(0.566)	(53.846)	(0.0330)	(0.0330)	(0.0330)	(0.0330)
Whether Hukou in urban	-0.750	-149.354***	0.0307	0.0307	0.0307	0.0307
	(0.500)	(45.758)	(0.0300)	(0.0300)	(0.0300)	(0.0300)
Whether ethnic minorities	-1.376	-59.204	-0.1802***	-0.1802***	-0.1802***	-0.1802***
	(0.929)	(77.577)	(0.0483)	(0.0483)	(0.0483)	(0.0483)
Whether religious believer	-0.729	-63.918	0.0463	0.0463	0.0463	0.0463
	(0.765)	(66.226)	(0.0355)	(0.0355)	(0.0355)	(0.0355)
Whether CPC member	-0.675	-100.061*	-0.0697**	-0.0697**	-0.0697**	-0.0697**
	(0.611)	(54.499)	(0.0327)	(0.0327)	(0.0327)	(0.0327)
ln_Income	1.436***	87.831***	0.0339**	0.0339**	0.0339**	0.0339**
	(0.102)	(8.029)	(0.0152)	(0.0152)	(0.0152)	(0.0152)
Socio-economic status	-1.268***	-142.126***	-0.1490***	-0.1490***	-0.1490***	-0.1490***
	(0.430)	(37.646)	(0.0207)	(0.0207)	(0.0207)	(0.0207)
Whether having pension	-1.249**	-147.423***	-0.0180	-0.0180	-0.0180	-0.0180
	(0.560)	(49.015)	(0.0257)	(0.0257)	(0.0257)	(0.0257)
Whether having medical insurance	-0.411	-47.166	0.0529	0.0529	0.0529	0.0529
	(0.935)	(82.779)	(0.0435)	(0.0435)	(0.0435)	(0.0435)
Whether married	0.905	78.682	-0.1340***	-0.1340***	-0.1340***	-0.1340***
	(0.612)	(53.287)	(0.0289)	(0.0289)	(0.0289)	(0.0289)
Family size	-0.039	8.537	-0.0070	-0.0070	-0.0070	-0.0070
	(0.151)	(13.136)	(0.0073)	(0.0073)	(0.0073)	(0.0073)
Number of children	0.137	22.072	0.0053	0.0053	0.0053	0.0053
	(0.257)	(22.143)	(0.0112)	(0.0112)	(0.0112)	(0.0112)
Number of houses	-0.751**	-55.493**	-0.0055	-0.0055	-0.0055	-0.0055
	(0.296)	(26.226)	(0.0155)	(0.0155)	(0.0155)	(0.0155)
Year dummy	Yes	Yes	Yes	Yes	Yes	Yes
Province dummies	Yes	Yes	Yes	Yes	Yes	Yes
Constant	4.550***	28.970***	3.8939***	3.8939***	3.8939***	3.8939***

	(0.378)	(4.332)	(0.3215)	(0.3215)	(0.3215)	(0.3215)
Observations	11655	11655	11655	11655	11655	11655

Supplementary Table 6. Penalized regression results

Model<br><br>Variable	(1)<br>Lasso<br>(10-fold CV)<br>Depression	(2)<br>Lasso<br>(20-fold CV)<br>Depression	(3)<br>Ridge<br>(10-fold CV)<br>Depression	(4)<br>Ridge<br>(20-fold CV)<br>Depression	(5)<br>Elastic Net<br>(10-fold CV)<br>Depression	(6)<br>Elastic Net<br>(20-fold CV)<br>Depression
Working_time	-0.00227	-0.00260	-0.00177	-0.00177	-0.00213	-0.00236
Working_time_ squared	0.00003	0.00004	0.00003	0.00003	0.00004	0.00004
No. of nonzero coefficients	46	46	50	50	47	47
λ	0.00119	0.00082	0.03481	0.03481	0.00544	0.00683
Out-of-sample R^2	0.1673	0.1672	0.1671	0.1671	0.1673	0.1672
CV mean prediction error	0.75608	0.75618	0. 75620	0.75627	0. 75608	0.75617
α					0.2	0.1
Observations	11655	11655	11655	11655	11655	11655

Supplementary Figure 1. Placebo test results

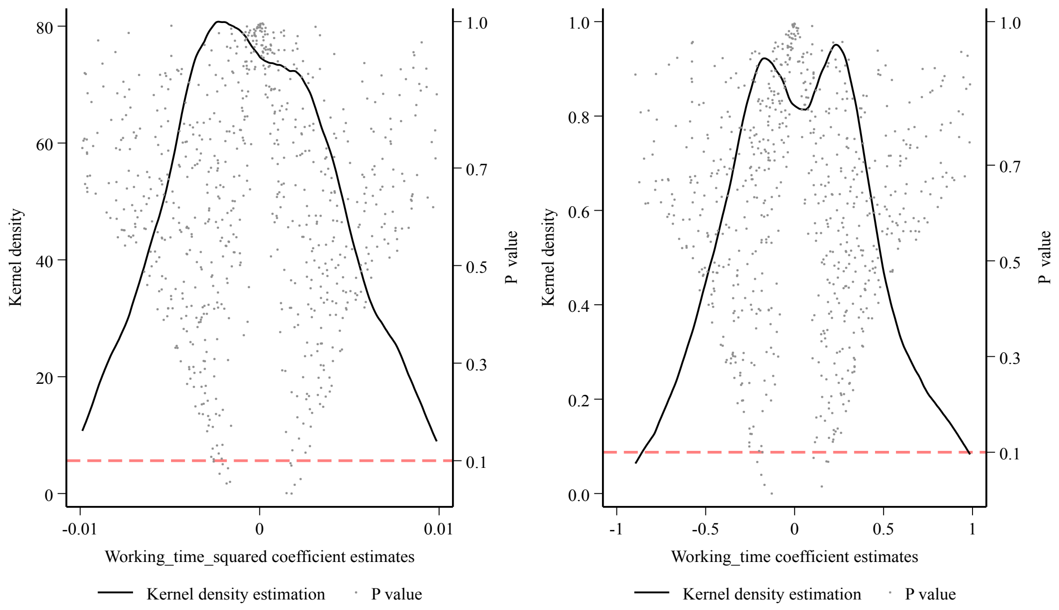

Note: The placebo test is carried out to clarify whether the U-shaped relationship between working time and depression is caused by other random factors omitted in the regressions. We randomly assign working hours 1000 times in CGSS and perform 2SLS estimations using these new samples. As illustrated in supplemental Figure1, the effects of working time and its quadratic term on mental disorders are not significant in almost all of the 1000 regressions, that is, their P values are larger than 0.1. This proves that the U-shaped relationship between working hours and depression is not caused by other omitted factors, relieving concerns about this endogeneity problem.

Supplementary Description of the Dataset

This paper uses a nationally representative observational dataset, which is the Chinese General Social Survey (CGSS). CGSS is in the world General Social Survey family, jointly carried out by Renmin University of China and Hong Kong University of Science and Technology. The sampling of CGSS is based on the multi-stage stratified design. The sampling stages are as follows: (1) PSUs are county-level units and there are 2762 PSUs in the sampling frame; (2) SSUs are community-level units (villages [cun] and neighborhood committees [ju wei hui]); (3) in selected SSU, 25 households (TSUs) are sampled with PPS method; (4) one eligible person aged 18-above is selected from each sampled household to serve as the representative. There are 43 Municipalities directly under the Chinese central government, provincial capital cities, and vice provincial cities in China. Comprehensive ranking by GDP, FDI and Education Level to these cities, the top 5 is Beijing, Shanghai, Tianjin, Guangzhou, and Shenzhen. CGSS treats these 5 cities as self-representative stratum. This stratum consists of 67 PSUs. The rest 2695 PSUs are comprehensively ranked with GDP per capital, urbanization rate, and population density and then are equally classified into 50 strata. Within each stratum, 2 PSUs will be selected with PPS method. In each selected PSU, 4 communities are sampled with PPS method. There are 80 communities in self-representative stratum and 400 communities in the rest 50 strata.

The national Survey Research Center at Renmin University of China (NSRC) organized Chinese Social Survey Network (CSSN), including 49 universities and provincial social science academies. Members of CSSN undertake the survey of the CGSS in their own provinces. The CGSS uses Computer Aided Personal Interviewing, and the average interview time is about one and a half hours. The CGSS has a set of strict quality control procedures, which cover pre-fieldwork, in-fieldwork, and after-fieldwork states. In pre-fieldwork stage, all supervisors must receive 40 hours training and finish 4 experimental interviews; all interviewers must receive 25 hours training and finish 3 experimental interviews. In in-fieldwork stage, all interviewers will be accompanied to interview by supervisor at least once. And the finished questionnaires must be 100% on site check and supervisors must do 40% the second day back interviewing. And in post-fieldwork stage, all interviews must 100% mail back interview and 40% telephone interview. In data input and coding stage, the data must be double input and double coding and there are several supervisors to check the double input and coding validation process.

The CGSS aims to collect quantitative data about (1) measures of social structure, its stability and change, (2) measures of quality of life, objective and subjective, and (3) measures of underlying mechanisms linking social structure and quality of life. The questionnaire of CGSS composes three kinds of modules: core module, topic module (rotation module), and additional module. The core module is annually repeated, which includes 152 variables. There are two kinds of the CGSS core module variables. One is the standard background variables, which include 71 variables. Another is the variables to trace social change trends. They are the rest 81 variables. The core module of the CGSS has 11 dimensions: Social demographic, health, lifestyle, migration, social attitude, class identity, political attitude and behavior, cognitive ability, labor market participation, social welfare, and family. The topic modules will be rotated every five years. There are one or two topic modules in the annual questionnaire. The topic modules aim to address important social issues. The additional modules include EASS module, ISSP module, and other ad hoc one-time modules. Some proposed topic modules also might be used as one-time additional modules. Questions in core module and

topic modules will be asked to all respondents. Questions in additional modules only have one-third or a half chance to be asked.

Above information is taken from http://cgss.ruc.edu.cn/English/Home.htm.
